# How research-based is our policy-making? Implementation of evidence-based treatments by State behavioral health systems, 2001-2012

**DOI:** 10.1186/1748-5908-10-S1-A40

**Published:** 2015-08-20

**Authors:** Eric J Bruns, Michael D Pullmann, Suzanne EU Kerns, Spencer Hensley, Ted Lutterman, Kimberly E Hoagwood

**Affiliations:** 1 Department of Psychiatry and Behavioral Sciences, University of Washington School of Medicine, Seattle, WA, 98102, USA; 2 NASMHPD Research Institute, Falls Church, VA, 22042, USA; 3 Department of Child Psychiatry, New York University School of Medicine, New York, NY, 10016, USA

## 

Despite the proliferation of evidence-based treatments (EBT), there is little evidence that they are successfully implemented in real world behavioral health systems, fueling an ongoing debate about whether and how systems should invest in infrastructure to support implementation of EBT and the use of data and research to inform policy. Ironically, however, there are few empirical studies of whether and how public systems are living up to the promise of data- and research-based decision-making. The current study used data from the National Association of State Mental Health Program Directors (NASHMPD) Research Institute's (NRI) longstanding effort to compile information on state mental health authorities to examine the degree to which state systems currently invest in EBT and data-driven decision-making, and how such investments have changed over the period from 2001-2012. The study focused on: (1) Rates of implementation of specific EBT programs; (2) Types of state-provided or-facilitated EBT training and workforce support; and (3) State investment in evaluation, data, and research use. Data originated from three NRI projects for which annual or semi-annual data relevant to the above research questions were available: the State Profiles System (SPS), the Revenues and Expenditures Study (R&E), and the Uniform Reporting System (URS). Results found that after an initial increase from 2001-2004, rates of implementation of both child and adult EBTs have been consistently flat for the last decade, with investment in EBTs for adults 2-6 times higher than for children's services. Data also show variability over time in the types of implementation supports being used, with states increasingly emphasizing EBT in contracts with providers, modification of IT systems, and "awareness building," and less on specific budget requests for EBT implementation or financial incentives for EBT use. Of concern, state investment in research and evaluation capacity and database integration efforts appear to be decreasing. Results of this ongoing research initiative are intended to strengthen the D&I knowledge base on the use of research findings in public systems, informing future national priorities and state efforts, promoting additional research, and serving as a baseline metric for reform efforts.

